# Effectiveness of School-based Education on HIV/AIDS Knowledge, Attitude, and Behavior among Secondary School Students in Wuhan, China

**DOI:** 10.1371/journal.pone.0044881

**Published:** 2012-09-07

**Authors:** Xiaohui Gao, Yu Wu, Yu Zhang, Naixing Zhang, Jie Tang, Jun Qiu, Xiaofang Lin, Yukai Du

**Affiliations:** 1 Department of Child and Maternal Health Care, School of Public Health, Tongji Medical College, Huazhong University of Science & Technology, Wuhan, Hubei Province, P. R. China; 2 Department of Social Medicine and Health Management, School of Public Health, Tongji Medical College, Huazhong University of Science and Technology, Wuhan, Hubei Province, P. R. China; 3 Guangzhou Medical College, Guangzhou, Guangdong Province, P.R. China; University of South Australia, Australia

## Abstract

**Background:**

Human immunodeficiency virus (HIV) and acquired immune deficiency syndrome (AIDS) are among the most complex health problems in the world. Young people are at high risk of HIV and AIDS infections and are, therefore, in need of targeted prevention. School-based HIV/AIDS health education may be an effective way to prevent the spread of AIDS among adolescents.

**Methods:**

The study was a school-based intervention conducted in three middle schools and two high schools in Wuhan, China, which included 702 boys and 766 girls, with ages from 11 to 18 years old. The intervention was a one-class education program about HIV/AIDS for participants. HIV/AIDS knowledge, attitude, and high-risk behaviors were investigated using an anonymous self-administered questionnaire before and after the education intervention. Chi-square test was used to compare differences before and after the intervention. Non-conditional logistic regression analysis was used to identify the factors that affect HIV/AIDS knowledge.

**Results:**

Misconceptions about basic medical knowledge and non-transmission modes of HIV/AIDS among all the students prevail. Approximately 10% to 40% of students had negative attitudes about HIV/AIDS before the intervention. After the intervention, all of the students had significant improvements in knowledge and attitude about HIV/AIDS (P<.05), indicating that educational intervention increased the students’ knowledge significantly and changed their attitudes positively. Logistic regression analyses indicated that before the intervention the students’ level of knowledge about HIV/AIDS was significantly associated with grade, economic status of the family, and attitudes toward participation in HIV/AIDS health information campaigns.

**Conclusions:**

HIV/AIDS education programs were welcomed by secondary students and positively influenced HIV/AIDS-related knowledge and attitudes. A systematic and long-term intervention among secondary school students must be conducted for the prevention of HIV.

## Introduction

### Global AIDS Epidemic

Human immunodeficiency virus (HIV) and acquired immune deficiency syndrome (AIDS) are among the most complex health problems of the 21^st^ century [1]. The year 2011 marked 30 years since the discovery of AIDS, which has claimed more than 25 million lives. More than 60 million people have been infected with HIV, and more than 90% of the cases occurred in developing countries [2]. In Asia, approximately 4.9 million (4.5 million–5.5 million) people were infected with HIV in 2009. Most national HIV epidemics appear to have stabilized [3]. The estimated number of children younger than 15 years, who are living with HIV increased significantly, from 140,000 (92,000–190,000) in 2005 to 160,000 (110,000–210,000) in 2009 [3].

### AIDS Epidemic in China

The first HIV case in China was reported in 1985 and the epidemic spread began in 1989 through injecting drug users. In rural areas of China, 31% provinces have reported cases of HIV [4], [5]. In 2009 according to the Chinese Ministry of Health there were 326,000 people living with HIV and of these 107,000 had AIDS [6]. In China the AIDS epidemic is complex with some populations effected more than other [6].

### HIV/AIDS in Hubei Province

Hubei province which lies in central China, in the middle of the Yangtze River with an area of 186,000 square kilometers and a population of 65 million. It is estimated that 45,000 people are HIV positive and that 83% of HIV cases are due to illegal commercial blood selling in 1990’s similar to the provinces of Yunnan, Guangxi, Anhui and Guangdong [4], [7], [8].

Young people are at high risk for HIV/AIDS infections [9]. Lack of knowledge about AIDS prevention makes them more vulnerable to HIV infection [5]. As a transitional step from children to adulthood, adolescence is a crucial period for fostering healthy attitudes and behaviors to protect people from diseases [10]. Thus, fostering healthy behaviors among adolescents may be more essential for the prevention of HIV/AIDS and high-risk behaviors in the general population [10]. Young people are valuable parts of the society, and they will be a powerful against the transmission of HIV in the future. Their opinions, attitudes, and behaviors play critical roles in constructing a compassionate social environment that is free from discrimination for people living with HIV/AIDS [11].

In China, schools are the primary locations where young people acquire knowledge and skills. School-based HIV/AIDS health education can be more efficiently operated and delivered than other programs that prevent the spread of AIDS [10].

Most of the previous studies on student s’ knowledge, attitudes, and behaviors (KAB) about HIV/AIDS in Hubei province were conducted among university students [4]. Secondary schools in Hubei province seldom carry out KAB research and provide systematic education to prevent HIV/AIDS. Therefore, we conducted an exploratory study in Hubei province on the existing levels and types of KAB of secondary students with regard to HIV/AIDS. The aim of this school-based study was to evaluate the effectiveness of an educational prevention program on HIV/AIDS, particularly in relation to the KAB of secondary school students, to provide theoretical and practical information for decision-makers.

## Methods

### Study Population

The study was conducted in Wuhan from March 1 to June 30, 2011. Wuhan is the capital city of Hubei province which occupies a land of 8,494, 41 square kilometers and has a population of 7,811,900 [12]. There were a number of 380 secondary schools with a population of 352,100 students in three districts (Hankou, Hanyang, and Wuchang) in Wuhan [13] and all of these schools had equal probability for selection. At last, three middle schools and two high schools located dispersed in Hankou district and Hangyang district were recruited using stratified random sampling. After being informed about the purpose of the study, all of the five schools agreed to participate. Students of the five schools and their parents/guardians were informed of the purpose of the study before the intervention. A consent letter was sent to them. The parents and the students then signed the informed consent together to indicate that they agreed to participate. Finally, all of the students and their parents/guardians agreed to participate in the intervention. Our total sample included 1,468 students (702 boys and 766 girls, about 0.4% of the secondary school students’ population in Wuhan) aged 11 to 18 years.

### Study Design

The study consisted of a quasi-experimental and school-based survey using a self-administered questionnaire for both genders.

### Data Collection Procedures

This study was divided into two stages: the baseline research and the intervention research. The test was administered at the end of each stage, before the intervention test, and after the intervention test using a self-administered questionnaire. The baseline research was conducted before the intervention began, and a similar research was conducted with the same group after the HIV/AIDS education. A total of 1,500 students (722 boys and 778 girls) completed the test before the intervention, and 1,468 students (702 boys and 766 girls) were successfully followed up after the intervention. Questionnaires were handed out and collected by trained investigators during school hours. The students’ names did not appear on the questionnaires.

### Intervention

The HIV/AIDS educational intervention consisted of two sections, namely a 30 minute lecture and a 15-minute promotional video, both of which focused on HIV/AIDS biology, epidemic situation in China, and all over the world, transmission and non-transmission modes, high-risk behaviors, preventive measures, and self-protect skills. Classes were taught by medical graduates. They had basic medical knowledge and were trained for seven days at the beginning of this study. Promotional video’s content was a complementary of teaching and it made HIV/AIDS knowledge easy to remember. Considering the different school situations, the forms of teaching included one lecture in class, one lecture in an amphitheater, and 45 minutes of broadcast through a closed circuit television.

### Survey Instrument

The questionnaire was based on the Adolescent AIDS knowledge scale by Zimet (1998) [14] and the request of knowledge about HIV/AIDS prevention for young people by the United Nations General Assembly Special Session [15]. The questionnaire consisted of 38 closed-ended questions, including 10 questions on socio-demographic information (sex, age, nationality, school year, academic level, father’s education and occupation, mother’s education and occupation and socioeconomic level), 18 questions on HIV/AIDS knowledge (HIV/AIDS transmission modes, non-transmission modes, and prevention, sources of the knowledge), 4 questions on HIV/AIDS attitude (attitude toward people living in HIV/AIDS and HIV/AIDS education needs), 5 questions on high-risk behaviors (drug of abuse, age at sexual initiation, and condom use ), and 1 question on source of HIV/AIDS knowledge. The tests before and after the intervention were the same questionnaire, however, the students were not required to fill out the part on high-risk behaviors after the intervention test.

Cronbach test resulted in a score of 0.77, which indicates a good reliability of the index and statistical significance.

### Statistical Analysis

Data were entered in duplicate, compared for errors, and analyzed using the Statistical Package for Social Sciences software (SPSS for Windows 15.0, SPSS Inc., Chicago, IL). We performed descriptive analysis on the socio-demographic characteristics and measured the rate of HIV/AIDS knowledge awareness. The Person chi-square test was used to compare the rate of HIV/AIDS knowledge awareness before and after the intervention as well as between middle and high school students. Finally, we built a non-conditional logistic regression analysis model to identify the factors that influence knowledge and awareness of HIV/AIDS situations. Significance level was set at 0.05, and the all tests were two-sided.

### Informed Consent and Ethical Considerations

The research protocol including the instruments was reviewed and approved by the Ethical Committee of the Medical Association of Tongji Medical College of Huazhong University of Science and Technology.

## Results

### Study Population

The response rate was 97.87% (1468/1500). The participants consisted of 877 middle school students and 591 high school students. The middle school included grade one (279 students) and grade two (598 students) levels. The high school included grade one (438 students) and grade two (153 students) levels. The mean age was 14.71 years (Mean ± SD: 14.71±1.49). Table 1 shows the socio-demographic characteristics of the students. Most of the parents of the participants’ finished junior secondary (40.26%) and senior secondary (38.56%). Most of the parents were self-employed and factory workers/laborers (father: 45.64%; mother: 37.60%). A total of 691 (78.79%) middle school students, and 455 (76.99%) high school students rated their family economic status as middle class. About 69.44% of the middle school students and 77.16% of the high school students had a middle scholastic achievement.

**Table 1:** Socio-demographic characteristics of study participants.

	Middle school		High school		Total	
	n	%	n	%	n	%
Total	877	59.74	591	40.26	1468	100.00
Gender						
Males	476	54.28	226	38.24	702	47.82
Females	401	45.72	365	61.76	766	52.18
Age (years)						
11∼14	768	87.57	1	0.17	769	52.38
15∼18	109	12.43	590	99.83	699	47.62
Nationality						
Han	867	98.86	583	98.65	1450	98.77
Ethnic minority	10	1.14	8	1.35	18	12.30
Father’s education status						
Illiterate/primary	67	7.64	28	4.74	95	6.47
Junior secondary	394	44.93	197	33.33	591	40.26
Senior secondary	312	38.58	254	42.98	566	38.56
College or above	104	11.86	112	18.95	216	14.71
Mather’s education status						
Illiterate/primary	118	13.45	61	10.32	179	12.19
Junior secondary	397	45.27	217	36.72	614	41.83
Senior secondary	291	33.18	213	36.04	504	34.33
College or above	71	8.10	100	16.92	171	11.65
Father’s occupation						
Worker/self-employed	393	44.81	277	46.87	670	45.64
Business/service person	112	12.77	68	11.51	180	12.26
Teacher/governmental/professional	117	13.34	122	20.64	239	16.28
Famer	9	1.03	2	0.34	11	0.75
Others	246	28.05	122	20.64	368	25.07
Mother’s occupation						
Worker/self-employed	311	35.46	241	40.78	552	37.60
Business/service person	183	20.87	92	15.57	275	18.73
Teacher/governmental/professional	101	11.52	100	16.92	201	13.70
Famer	5	0.57	2	0.34	7	0.48
Others	277	31.58	156	26.40	433	29.49
Economic status						
High	147	16.76	92	15.57	239	16.28
Middle	691	78.79	455	76.99	1146	78.07
Low	39	4.45	44	7.45	83	5.65
Scholastic achievement						
High	136	15.51	72	12.18	208	14.17
Middle	609	69.44	456	77.16	1065	72.55
Low	132	15.05	63	10.66	195	13.28

### HIV/AIDS Knowledge Situation before and after Intervention

Before the intervention test, the students’ answers to the test pertaining to basic medical knowledge of HIV/AIDS indicated that the middle school students had a low rate of awareness, except in two questions such as “AIDS is an infected disease” (84.72%) and “AIDS can be prevented” (83.35%). A kind of intermediate level of knowledge regarding HIV/AIDS among high school students was found before intervention. After the intervention test, the answers revealed an increase in basic medical knowledge of HIV/AIDS, which can be attributed to the intervention. Significant differences before and after the intervention tests for all questions among in middle and high school students were discovered (Table 2).

**Table 2:** Percentage and comparison of HIV/AIDS knowledge before and after intervention by secondary school students (n = 1,468) in Wuhan, China.

Variable	Middle School		High School		*P_1_*	*P_2_*	*P_3_*	*P_4_*
	Before intervention	After intervention	Before intervention	Afterintervention				
Basic medical knowledge								
Does AIDS caused by a kind of virus?	302(34.44)	793(90.42)	373(63.11)	532(90.02)	<.0001	<.0001	<.0001	0.7975
A person can be known infected from appearance	392(44.70)	726(82.78)	407(68.87)	507(85.79)	<.0001	<.0001	<.0001	0.1236
AIDS is an infected disease	743(84.72)	843(96.12)	521(88.16)	568(96.11)	<.0001	<.0001	0.0620	0.9885
AIDS can be totally cured at present	341(38.88)	775(88.37)	397(67.17)	558(94.42)	<.0001	<.0001	<.0001	<.0001
AIDS can be prevented	731(83.35)	810(92.36)	510(86.29)	543(91.88)	<.0001	0.0011	0.1263	0.7360
Transmission knowledge								
Blood can transmit HIV	699(79.70)	838(95.55)	564(95.43)	578(97.80)	<.0001	0.0231	<.0001	0.0223
Sharing injection needle of an infected person can be infected	695(79.25)	843(96.12)	546(92.39)	572(96.79)	<.0001	0.0004	<.0001	0.5049
Shaving/tattooing/getting ear pierce with unsterilized toolscan be infected	457(52.11)	776(88.48)	389(65.82)	558(94.42)	<.0001	<.0001	<.0001	<.0001
Donating or transfusing untested blood through non-formal way can be infected	664(75.71)	822(93.73)	547(92.55)	573(96.95)	<.0001	0.0005	<.0001	0.0053
An infected pregnant woman can infect her unborn baby	597(68.07)	843(96.12)	532(90.02)	571(96.62)	<.0001	<.0001	<.0001	0.6228
Having unprotected sex with infected person can be infected	653(74.46)	820(93.50)	547(92.55)	570(96.45)	<.0001	0.0013	<.0001	0.0136
Breast milk can transmit HIV	501(57.13)	808(92.13)	348(58.88)	516(87.31)	<.0001	<.0001	0.5039	0.0023
Non-transmission knowledge								
Hugging/kissing/shaking hands with an infected personcan be infected	325(37.06)	667(76.05)	481(81.39)	554(93.74)	<.0001	<.0001	<.0001	<.0001
Sharing toilet seat/swimming pool with an infected personcan be infected	254(28.96)	650(74.12)	394(66.67)	542(91.71)	<.0001	<.0001	<.0001	<.0001
Sharing cups/dinner set/bedding/tools of an infected personcan be infected	172(19.16)	533(60.78)	398(67.34)	530(89.68)	<.0001	<.0001	<.0001	<.0001
Mosquitoes can transmit HIV	164(18.70)	760(86.66)	158(26.73)	550(93.06)	<.0001	<.0001	0.0003	0.0001
Study in the same classroom with an infected personcan be infected	415(47.32)	706(80.50)	490(82.91)	541(91.54)	<.0001	<.0001	<.0001	<.0001
Coughing/sternutation can transmit HIV	174(19.84)	583(66.48)	367(62.10)	540(91.37)	<.0001	<.0001	<.0001	<.0001

Note:

*P_1_*: before intervention vs after intervention in middle school students’ HIV/AIDS knowledge awareness rate;

*P_2_*: before intervention vs after intervention in high school students’ HIV/AIDS knowledge awareness rate;

*P_3_*: middle school vs high school before intervention about students’ HIV/AIDS knowledge awareness rate;

*P_4_*: middle school vs high school after intervention about students’ HIV/AIDS knowledge awareness rate;

As to the manners of transmission of HIV/AIDS, we found that before intervention, more than 65% of the middle school students and 90% of the high school students identified three modes of transmission, namely sexual, blood transfusions, and mother-to-child transmission. We found that 52.11% of middle school students and 65.82% of high school students knew that “shaving/tattooing/getting ear pierce with unsterilized tools can cause infection”. After the intervention, the rate of knowledge about the manner of transmission increased to more than 85% and 90% in both groups respectively with statistically significant differences (Table 2).

However, the participants had several misconceptions about the non-transmission way. A large proportion of middle and high school students (18.70% and 26.73%, respectively) believed that mosquitoes can spread the virus. Confusion also existed as to non-risk behaviors, especially casual contact with people who live with HIV/AIDS (sharing cups/dinner set/bedding/tools, sharing toilet seat/swimming pool). After the education intervention, the rate of awareness of the middle school students increased into a moderate level, whereas the rate of awareness of the high school students rose to a high level with statistically significant differences in both groups (Table 2).

Before the intervention, the high school students had a higher level of knowledge about HIV/AIDS than the middle school students, with statistically significant differences except in three questions, namely, “AIDS is an infected disease” (*P* = 0.0620), “AIDS can be prevented” (*P* = 0.1263), and “Breast milk can transmit HIV” (*P* = 0.5039) (Table 2).

After the intervention, the middle school students showed a similar level of basic medical knowledge about HIV/AIDS like the high school students, except in one question, namely, “AIDS can be totally cured at present” (*P*<.0001). As to the participant s’ knowledge of HIV transmission, we found that some of the middle school students still misunderstood the blood and sexual ways. The participant s’ answers to the three questions pertaining to shaving/tattooing/getting ear pierce with unsterilized tools, donating or transfusing untested blood through non-formal way, and having unprotected sex with infected person, revealed that the middle school students did not achieve the same level of knowledge as the high school students. With regard to their knowledge of non-transmission of HIV, the middle school students obtained a higher rate before intervention but still lower compared with the rate of the high school students, indicating significant differences in all of the questions (Table 2).

### Knowledge Sources of HIV/AIDS

The sources of knowledge on HIV/AIDS were designed into multiple choices. Television/broadcast was reported as the major source of information about HIV/AIDS (1003, 68.32%). Newspaper/books/magazines (996, 67.85%), internet (705, 48.02%), lectures (n = 593, 40.40%), school education (525, 35.76%), doctors (493, 33.58%), friends/classmates (441, 30.04%), parents (412, 28.07%), and watching video/perform (399, 27.18%) were the other sources of information. Among these sources, television/broadcast (821, 55.93%) was reported as their favorite.

### Attitude toward PLHA

Table 3 shows that before the intervention test, a total of 537 (61.23%) middle school students and 406 (68.70%) high school students said that they would like to help people living with HIV/AIDS. This rate increased after the intervention test to 660 (75.26%) and 496 (83.93%), respectively (*P*<.05). This means that 80.96% of the middle school students and 87.48% of the high school students would like to take care of their classmates/families if they were infected with HIV, whereas only 72.06% and 81.73% of middle school and high school students, respectively, expressed a similar response before the intervention. As to educational needs, more than 80% of the students believed that the school should upgrade the curricula about HIV/AIDS before and after the intervention. Significant differences between the initial and final- intervention tests were observed among the middle school students (*P*<.05). However, no significant differences were observed among the high school students (*P* = 0.1248). With regard to their participation in HIV/AIDS knowledge propaganda, we discovered that a total of 710 middle school students and 489 high school students wanted to try. The rate reached 86.32% and 87.31%, respectively, after our education and intervention (*P*<.05) (Table 3).

**Table 3:** Students’ HIV/AIDS attitude rate comparison between before and after intervention.

Variable	Middle School			High School		
	Before intervention	After intervention	*P*	Before intervention	After intervention	*P*
Would you like to help PLHA?						
Yes	537(61.23)	660(75.26)	<.0001	406(68.70)	496(83.93)	<.0001
No	340(38.77)	217(24.74)		185(31.30)	95(16.07)	
Would you like to take care of your families or classmates if they wereinfected by HIV?						
Yes	632(72.06)	710(80.96)	<.0001	483(81.73)	517(87.48)	0.0005
No	245(27.94)	167(19.04)		108(18.27)	74(12.52)	
School education should increase curricula or not						
Yes	744(84.83)	797(90.88)	<.0001	514(86.97)	526(89.00)	0.1248
No	22(2.51)	12(1.37)		16(2.71)	7(1.18)	
I do not care	111(12.66)	68(7.75)		61(10.32)	58(9.81)	
Would you like to participate in HIV/AIDS health information campaigns?						
Yes	710(80.96)	757(86.32)	<.0001	489(82.74)	516(87.31)	0.0010
No	167(19.04)	120(13.68)		102(17.26)	75(12.69)	

### High-risk Behaviors about HIV/AIDS

Before the intervention test, 27 (1.84%) students reported having sexual relations. The sexual initiation age range was 11 years to 18 years old. In addition, 20 (74.07%) students among the sexually active students reported knowing how to protect themselves during the sexual encounter. Twenty-two students (1.50%) reported drug and substance abuse, and six of these students shared injection needles with others. We did not measure the behavior change immediately after the intervention because people needed time to reform.

### Factors Associated with Knowledge of HIV/AIDS

The test on knowledge of HIV/AIDS consisted of 18 yes/no questions. One correct answer receives one point, with a total of 18 points, which were transmitted to a 100 score. We categorized the score into three levels, that is, low (≤60), medium (61–79), and high (≥80) based on the scores. A non-conditional logistic regression model was built. The outcome variable was the level of knowledge about HIV/AIDS and the predictive variables including socio-demographic variables and HIV/AIDS attitude variables before intervention. Table 4 shows all of the effects entered into the model. Only those results that are statistically significant (*P*<.05) are included in the discussion below. The results indicate that a higher level of knowledge of HIV/AIDS is significantly associated with a higher grade (*OR* = 2.288, 95% *CI* = 2.097, 2.496). Low family economic status was a negative factor for the level of the students’ knowledge of HIV/AIDS (*OR* = 0.746, 95% *CI* = 0.613, 0.907). The students who did not want to participate into the HIV/AIDS knowledge publicity before intervention had a lower level of knowledge of HIV/AIDS than those who wanted to participate (*OR* = 0.732, 95% *CI* = 0.545, 0.983).

**Table 4:** Non-conditional logistic regression analysis with secondary school students’ HIV/AIDS knowledge influence factors in Wuhan, China.

Effect	*Estimate*	*Standard Error*	*Wald Chi-Square*	*Pr > ChiSq*	*Point Estimate*	*95% Wald Confidence Limits*
Gender	−0.1880	0.1058	3.1586	0.0755	0.829	0.673–1.020
Grade	0.8276	0.0443	348.4179	<.0001	2.288	2.097–2.496
Father’s occupation						
Worker/self-employed vs Famer	0.0102	0.1488	0.0047	0.9452	1.237	0.348–4.403
Business person/services vs Famer	−0.00456	0.1793	0.0006	0.9797	1.219	0.334–4.456
Teacher/governmental/professional vs Famer	0.4048	0.1684	5.7760	0.0162	1.836	0.507–6.643
Others vs Famer	−0.2076	0.1586	1.7126	0.1906	0.995	0.278–3.567
Economic status	−0.2932	0.0999	8.6112	0.0033	0.746	0.613–0.907
Whether want to help PLHA(before intervention)	−0.2232	0.1161	3.6972	0.0545	0.800	0.637–1.004
Whether want to take care PLHA(before intervention)	−0.2218	0.1302	2.9032	0.0884	0.801	0.621–1.034
School HIV/ADIS education needs(before intervention)						
Do not need vs need	−0.2804	0.2352	1.4220	0.2331	0.578	0.291–1.150
I do not care vs need	0.0129	0.1574	0.0068	0.9345	0.775	0.548–1.098
Whether want to participate into HIV/AIDS health information campaigns (before intervention)	−0.3115	0.1504	4.2911	0.0383	0.732	0.545–0.983

## Discussion

This study revealed an imbalance knowledge or awareness of the situation of people infected with HIV/AIDS among secondary school students in Wuhan. As to the basic medical knowledge, 80% of the students knew before the invention that “AIDS is an infectious disease but can be prevented” This rate increased to more than 90% after our education. The awareness rate of the students on these questions was higher than the rate of awareness of other samples in other previous studies [16]. However, no differences among the middle and high school students before and after the intervention tests were found, indicating that this point of the basic medical knowledge of HIV/AIDS was known widely before and was easy to recognize after intervention. However, only 38.88% of the middle school students and 67.17% of the high school students knew that no cure exists for AIDS. This finding indicated that although the students may have been poorly informed about a cure or vaccine because HIV/AIDS is not a common occurrence in their daily study and lives, the lack of knowledge could have resulted from the complexity of information on the treatments and cures of AIDS from conflicting media coverage [17].

As for transmission and non-transmission modes, the high school students had a higher level of knowledge than the middle school students. Very few knew that “mosquitoes cannot transmit HIV” (26.73%). Similarly, they only had intermediate levels of knowledge before our education. This result is in agreement with the levels of knowledge of HIV/AIDS found among the Mexican public school students [18] and the Nigerian secondary school students [19].

After the intervention, both the middle and high school students showed an increased rate of awareness. After our intervention, the middle school students achieved the same basic medical and transmission mode level of knowledge as the high school students, expect in relation to the question on non-transmission part. The results of the study suggest that the high school students understood the contents of the educational materials because they had learned some relevant knowledge from their biology class and from other relevant curricula. The contents of our intervention materials can be richer and deeper for high school students in the future. However, the middle school students were seldom given knowledge about HIV/AIDS in their primary grade. Some parts of the test on HIV/AIDS, such as non-transmission modes, need additional time to explain.

As reported elsewhere, television/broadcast was the principal source of knowledge about HIV/AIDS [1]. Unfortunately, TV and broadcast sources had no negative effect on knowledge of HIV/AIDS [17], [20]. The Internet was also an important source because adolescents were active Internet users. Our finding is similar to the findings in a previous study [21]. Our finding that schools and parents were not the main sources of knowledge about HIV/AIDS indicate that teachers were not sufficiently conscious about HIV/AIDS prevention. Parents have difficulty in speaking openly about HIV/AIDS with their children although talking about HIV/AIDS with parents improved the students’ knowledge about AIDS [20].

### Attitude

Most students believed that being infected with HIV would never happen to them because HIV/AIDS is a social problem and not a school problem. Thus, before our education, few students cared about people with HIV/AIDS and whether those who are infected really need their help. However, after the intervention, a large number of middle and the high school students changed their negative attitude to a positive one, specifically in relation to these two issues. These results indicate that our education dispelled the students’ fear of people with HIV/AIDS, thereby enabling them to established friendly attitudes toward HIV-infected people. This result is parallel to the results found in other studies in Ukraine [22] and Nigeria [23]. Before the intervention test, the students were asked if they would be interested in a new curriculum. More than 80% of the middle school and high school students indicated that they would like to take part in learning and sharing their knowledge of HIV/AIDS. The results suggest that the students appreciated the educational activities. In fact, they perceived the intervention as valuable and enjoyable, although they need a systematic intervention [11], [24], [25].

### Behaviors

The students who participated in our research, especially the middle school students, were ashamed to talk about sex and drug abuse. Most of them thought that the questions about sexual activities and use of illegal drug and substance in the test questionnaires before intervention were jokes. Hence, their answers were dubious and biased. Teachers believed that their students may be have puppy love but would never have sex, thus teachers were reluctant to talk about safe sex [26], [27]. School principals were also worried that talking about sex would disrupt the students’ mental health and negatively influence their school works. Hence, future studies need to postpone relevant intervention until the end of a systematic long-term education programs in the future [28].

Our regression analysis showed that before our intervention, the level of the students’ HIV/AIDS knowledge was significantly associated with the students’ grade, economic status of the family, and attitude toward “whether they want to participate in HIV/AIDS health information campaigns.” Our intervention is consistent with the results of a similar study conducted in Flemish secondary schools [29].

### Conclusions

In conclusion, this study suggests that educational programs on HIV/AIDS prevention are effective and beneficial to secondary school students. HIV/AIDS education will be more successful if education is carried out using continuous and long-term strategies with realistic objectives [1], [22]. Decision-makers as well as school headmasters and teachers should realize that school education is an effective solution to prevent the spread of the HIV/AIDS epidemic [30]–[32]. Relevant curricula should be developed every semester and topics in middle school should be different from those in high school depending on the knowledge structures and the perception abilities of the students. This education program should be extended to more middle schools to multiply the effects of providing opportunities to equip students with factual information on HIV/AIDS [22], [33].

### Limitations

Several limitations of our study need to be discussed. First, the population of the study only included Grade One and Two students from each selected school because of the heavy course load of the Grade Three students. In addition, this study only considered adolescents in school instead of the same aged out-of-school youths. Therefore, the results cannot be used to make a generalization about out-of-school youths. The program needs much further research to prove that it can be transferred elsewhere. The honesty of some responses should also be interpreted with caution, especially those that pertain to questions about sex activities and drug and substance abuse, because the data were self-reported by the students.

Another limitation is that sex is a sensitive topic in school. We were only allowed to determine the students’ high-risk behaviors through the questionnaire before the intervention test. We were not allowed to talk about the use of condom other sexual activities during the intervention. We unknown if the reported change in attitude resulted in behavior change at present.

Besides, lack of control group and no follow-up are also this study’s limitations. Our sample was representative but cannot avoid sample bias.

Future research needs to focus on long-term and multi-stage interventions on the knowledge of secondary school students about HIV/AIDS, together with training on self-protection skills. School leaders and teachers should also be actively interacted with students to ensure that additional health topics can be carried out effectively.

Education administration should strengthen the ability to integrate program into curriculum.
